# Targeted Inhibition of Oncogenic microRNAs miR-21, miR-17, and miR-155 Suppresses Tumor Growth and Modulates Immune Response in Colorectal Cancer

**DOI:** 10.3390/pharmaceutics18010122

**Published:** 2026-01-18

**Authors:** Olga Patutina, Aleksandra Sen’kova, Svetlana Miroshnichenko, Mona Awad, Oleg Markov, Daniil Gladkikh, Innokenty Savin, Ekaterina Seroklinova, Sergey Zhukov, Maxim Kupryushkin, Mikhail Maslov, Valentin Vlassov, Marina Zenkova

**Affiliations:** 1 Institute of Chemical Biology and Fundamental Medicine, Siberian Branch of the Russian Academy of Sciences, 630090 Novosibirsk, Russia; patutina@1bio.ru (O.P.); senkova_av@1bio.ru (A.S.); sveta-mira@yandex.ru (S.M.); mona.awad.edu@gmail.com (M.A.); markov_ov@1bio.ru (O.M.); medulla35@gmail.com (D.G.); savin_ia@1bio.ru (I.S.); seroklinovaekatherinag@gmail.com (E.S.); jsvbsasp@yandex.ru (S.Z.); kuprummax@1bio.ru (M.K.); vvv@niboch.nsc.ru (V.V.); 2 Faculty of Natural Sciences, Novosibirsk State University, 630090 Novosibirsk, Russia; 3 Lomonosov Institute of Fine Chemical Technologies, MIREA—Russian Technological University, 119571 Moscow, Russia; mamaslov@mail.ru

**Keywords:** miRNA, anti-miRs, mesyl phosphoramidate oligonucleotides, thymus atrophy, immunosuppression, MDSC, colorectal cancer

## Abstract

**Background and Objectives:** Aggressive cancer development is characterized by rapid tumor growth and progressive immune dysfunction. Tumor-derived microRNAs (miRNAs) emerge as master regulators of both malignant transformation and immune evasion, making them promising therapeutic targets. Using the highly aggressive CT-26 peritoneal adenomatosis model, this study explored the potential of selective miRNA inhibition to simultaneously suppress tumor growth and overcome immunosuppression. **Methods and Results:** Our results revealed that inhibition of miR-155, miR-21, and miR-17 by methylsulfonyl phosphoramidate (mesyl) oligonucleotides exhibited markedly different therapeutic profiles. miR-155 inhibition demonstrated minimal efficacy. miR-21 suppression provided early tumor regression and prevented cancer-associated thymic atrophy, translating into extended survival. miR-17 inhibition displayed delayed but superior tumor growth inhibition, significantly reducing pathologically elevated polymorphonuclear myeloid-derived suppressor cell (MDSC) populations, and nearly doubled animal lifespan. Combination therapy targeting all three miRNAs integrated these complementary mechanisms, maintaining consistent anti-tumor efficacy across early and late stages while providing thymic protection and MDSC reduction. Importantly, therapeutic responses in vivo substantially exceeded predictions based on in vitro tumor cell proliferation and motility measurements, revealing critical contributions of systemic immunomodulation. **Conclusions:** These findings demonstrate that miRNA inhibition reshapes tumor–immune interactions, positioning anti-miRNA therapeutics as immunomodulatory agents for effective colorectal cancer treatment.

## 1. Introduction

Among the multiple factors maintaining intestinal homeostasis, gene regulatory networks play a critical role in coordinating epithelial renewal, immune surveillance, and barrier function. MicroRNAs (miRNAs), small non-coding RNAs of 18–24 nucleotides, have emerged as master regulators of intestinal homeostasis, controlling epithelial cell differentiation, proliferation, and immune responses through post-transcriptional regulation of target mRNAs [1,2,3]. Dysregulation of miRNA expression disrupts normal intestinal function, creating conditions conducive to chronic inflammation, immune evasion, and ultimately malignant transformation [4,5].

Colorectal cancer (CRC), arising from disrupted intestinal homeostasis, is characterized by both malignant epithelial transformation and profound alterations in the tumor microenvironment, including chronic inflammation, metabolic reprogramming, angiogenesis, and severe immunosuppression [6,7,8]. The intricate interplay between these pathological processes necessitates therapeutic strategies capable of simultaneously targeting multiple oncogenic and immunosuppressive mechanisms, with multitargeted approaches representing particularly promising therapeutic avenues [9,10].

A distinctive feature of miRNA function is the capacity to regulate multiple target genes and entire signaling pathways simultaneously, positioning individual miRNA-targeting agents or their combinations as potentially powerful tools for multifunctional anti-tumor therapy [11,12,13]. In CRC, aberrant miRNA expression patterns are consistently associated with tumor initiation, progression, metastasis, and therapeutic resistance. Numerous miRNAs have been identified as key players in CRC pathogenesis, including miR-143/145, miR-34a, miR-146a, and miR-200 family members, which regulate critical oncogenic pathways including Wnt/β-catenin, PI3K/AKT, MAPK, and TGF-β signaling, thereby controlling cell proliferation, apoptosis, invasion, and epithelial-mesenchymal transition [5,14,15,16]. Among these miRNAs, miR-21, miR-155, and the miR-17-92 cluster have been most extensively implicated in CRC pathogenesis. miR-21 promotes tumor cell proliferation, invasion, and chemoresistance by targeting tumor suppressors including PTEN, PDCD4, and SMAD7 [17,18]. miR-155 drives CRC progression and contributes to microsatellite instability by targeting DNA mismatch repair proteins MLH1, MSH2, and MSH6 [19,20,21]. The miR-17-92 cluster enhances tumor cell proliferation and survival through coordinated targeting of multiple tumor suppressor proteins including PTEN, RND3, and E2F family members [5,22,23].

Importantly, miR-21 and miR-155 exert pleiotropic oncogenic effects, critically promoting tumor-associated immunosuppression through regulation of myeloid-derived suppressor cells (MDSCs) [24,25]. MDSCs represent a heterogeneous population of immature myeloid cell precursors that accumulate in tumor-bearing hosts and constitute a major barrier to effective anti-tumor immunity [9,26]. These cells exert potent immunosuppressive effects through multiple mechanisms: upregulation of iNOS and Arg1 impairs T-cell proliferation through L-arginine depletion; generation of reactive oxygen species; and secretion of immunosuppressive cytokines including IL-10 and TGF-β modulates the ratio of regulatory to effector immune cells. Furthermore, MDSCs promote the differentiation of immunosuppressive M2-type tumor-associated macrophages [10,27]. Specifically, miR-155 regulates MDSC-mediated immunosuppression through dual mechanisms—promoting their accumulation in the tumor microenvironment and enhancing suppressive function via SOCS-1 repression and facilitation of regulatory T-cell generation [28,29]. Hypoxia-induced glioma-derived exosomal miR-21 and miR-10a directly inhibit RORA and PTEN, respectively, thereby promoting MDSC expansion and enhancing their suppressive activity [30]. Furthermore, it was confirmed that tumor-derived miR-21 and miR-155 reprogram myeloid cells toward immunosuppressive MDSCs that potently inhibit CD8^+^ T-cell responses [31,32]. The miR-17-92 cluster is implicated in immune regulation, particularly enhancing type 1 T-cell differentiation and cytotoxic function [33,34,35], whereas its direct role in tumor-mediated MDSC expansion and immunosuppression remains less characterized compared to miR-21 and miR-155.

The combination of direct oncogenic activity in malignant cells with immunosuppressive functions in the tumor microenvironment makes miR-21, miR-155, and miR-17 particularly attractive targets for multifunctional anti-tumor therapy. To investigate the therapeutic potential of inhibiting these miRNAs, we used recently developed methylsulfonyl phosphoramidate (mesyl or µ)-modified anti-sense oligonucleotides (µ-ONs) [36]. The mesyl modification represents a promising advancement in therapeutic oligonucleotide chemistry, providing an attractive alternative to widely used phosphorothioates. The mesyl backbone confers distinctive advantages over phosphorothioate and other existing chemistries: superior nuclease resistance, enhanced RNase H1 recruitment efficiency, reduced non-specific protein interactions minimizing off-target effects and systemic toxicity, and functional sufficiency without requiring additional chemical modifications for therapeutic efficacy. Our previous studies demonstrated that µ-ONs provide potent suppression of target miRNAs in tumor cells, resulting in pronounced anti-oncogenic effects across various cancer models [11,36,37]. Importantly, these studies provided compelling evidence for the efficacy of combinatorial approaches: paired µ-ON combinations targeting miR-21, miR-17, and miR-155 synergistically suppressed B16 melanoma cell proliferation and migration in vitro, while the triple combination exhibited highly potent metastasis inhibition in the B16 model and substantial tumor growth suppression in RLS_40_ lymphosarcoma in vivo [24]. These prior studies focused primarily on direct anti-tumor effects without systematic evaluation of immunomodulatory consequences. In the present study, we extended this combinatorial strategy to the aggressive CT-26 peritoneal adenomatosis model characterized by rapid tumor progression and severe immunosuppression, evaluating the therapeutic potential of targeting miR-21, miR-155, and miR-17 both as monotherapies and in triple combination. The study was designed to assess whether individual miRNA suppression could serve as a multitargeted therapeutic tool by simultaneously affecting tumor growth and immune function, and whether combinatorial targeting provides enhanced therapeutic benefit.

## 2. Materials and Methods

### 2.1. Oligonucleotide Synthesis

**Figure 1:**
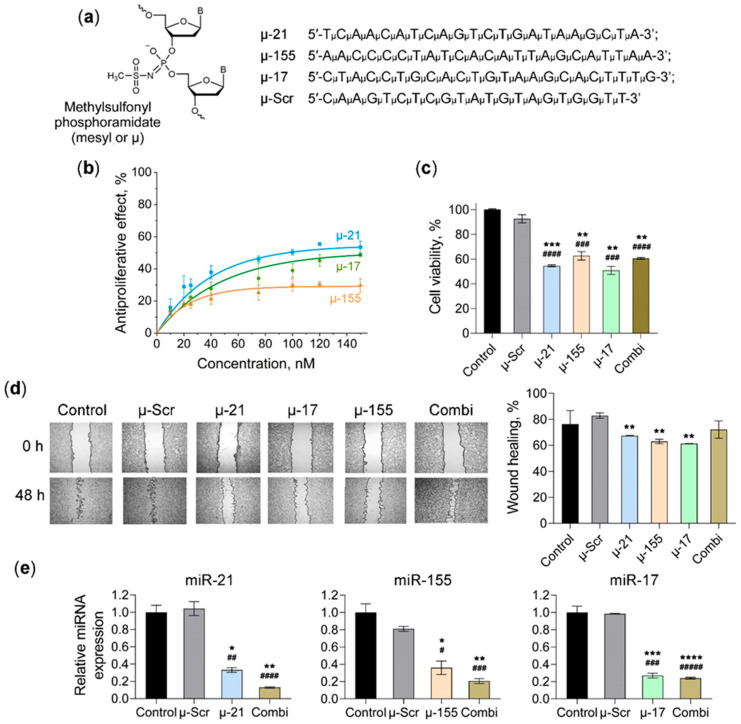
Biological performance of miRNA-targeted μ-oligonucleotides in CT-26 carcinoma cells. (**a**) Structure of the methylsulfonyl phosphoramidate (mesyl or μ) modification of the internucleotidic phosphate group and sequences of oligonucleotides targeting miR-21 (µ-21), miR-155 (µ-155), miR-17 (µ-17), and a non-targeting control (µ-Scr). (**b**) Dose-dependent anti-proliferative effects of µ-ONs 72 h post-transfection. (**c**) Viability of CT-26 cells 72 h post-transfection with individual μ-ONs or their triple combination (Combi). (**d**) Anti-migratory effect of µ-ONs or Combi assessed by wound-healing assay. Representative images (left panel) showing scratch boundaries (black lines) at 0 h and 48 h post-scratching (4× magnification) and degree of wound healing (right panel) after 48 h. (**e**) Stem-loop PCR data showing miRNA expression levels 48 h after cell transfection with a specific μ-ON or Combi. miR-21, miR-17, and miR-155 levels were normalized to U6 snRNA expression. Control—untreated cells; μ-Scr, μ-21, μ-17, and μ-155—cells transfected with control or miRNA-targeted μ-ONs (120 nM); Combi—cells transfected with a combination of three μ-oligonucleotides (40 nM each). Transfection was performed using cationic liposomes 2X3-DOPE. Data represent mean ± SEM of ≥3 independent experiments. The data were analyzed using Student’s *t*-test. ^#^, ^##^, ^###^, ^####^, ^#####^—significant differences from Control with *p* < 0.05, *p* < 0.01, *p* < 0.001, *p* < 0.0001, and *p* < 0.00001, respectively. *, **, ***, ****—significant differences from μ-Scr with *p* < 0.05, *p* < 0.01, *p* < 0.001, and *p* < 0.0001, respectively.

Fully modified methylsulfonyl phosphoramidate oligonucleotides (μ-ONs) were synthesized by automated solid-phase synthesis using phosphoramidite chemistry as previously described [37]. Following synthesis, oligonucleotides were deprotected and purified by reversed-phase high-performance liquid chromatography (RP-HPLC) using an Agilent 1260 HPLC system (Agilent Technologies Inc., Santa Clara, CA, USA). Oligonucleotide purity and identity were verified by mass spectrometry. Oligonucleotide sequences targeting miR-21 (μ-21), miR-155 (μ-155), and miR-17 (μ-17), as well as a non-targeting scrambled control oligonucleotide (μ-Scr), are listed in Figure 1a. Cyanine 5.5 (Cy5.5) was attached to µ-21 through a 3′-aminohexyl linker according to the manufacturer’s protocol, using the Cy5.5 N-hydroxysuccinimide ester (Biotech Industry Ltd., Moscow, Russia) in 0.1 M Tris buffer (pH 8.4).

### 2.2. Cell Transfection

CT-26 murine colorectal adenocarcinoma cells (Cell Culture Bank of the Blokhin National Medical Oncology Research Center, Moscow, Russia) were transfected with oligonucleotides using 2X3-DOPE (1:1 mol. ratio) cationic liposomes as previously described [38,39]. Lipoplexes were formed at an N/P ratio of 4/1 (molar ratio of nitrogen in cationic lipids to phosphorus in oligonucleotide backbone). 2X3-DOPE liposomes and oligonucleotides were separately diluted in equal volumes of Opti-MEM (Thermo Fisher Scientific, Waltham, MA, USA), mixed, and incubated for 20 min at room temperature to allow complex formation. For transfection, a culture medium was replaced with serum- and antibiotic-free IMDM (Iscove’s Modified Dulbecco’s Medium, Sigma-Aldrich, St. Louis, MO, USA). Lipoplexes were added to cells and incubated for 4 h at 37 °C in 5% CO_2_. Subsequently, the medium was replaced with complete DMEM (Dulbecco’s Modified Eagle Medium, Sigma-Aldrich, St. Louis, MO, USA) containing 10% FBS (Gibco, Paisley, Scotland, UK) and 1% antibiotic-antimycotic solution (10,000 μg/mL streptomycin, 10,000 IU/mL penicillin, 25 μg/mL amphotericin B, MP Biomedicals, Seven Hills, NSW, Australia), and cells were cultured for 24–72 h under standard conditions.

### 2.3. Cell Viability Test

Antiproliferative activity of μ-ONs was evaluated using the MTT assay. CT-26 cells were seeded in 96-well plates at a density of 7 × 10^3^ cells per well a day before transfection. Cells were transfected with individual oligonucleotides at concentrations ranging from 10 to 150 nM or with a triple combination at a total concentration of 120 nM (40 nM of each oligonucleotide) as described above. At 72 h post-transfection, MTT solution (5 mg/mL; Sigma-Aldrich, USA) was added to each well at a 1:10 (*v*/*v*) dilution and incubated for 3 h at 37 °C in 5% CO_2_. Following incubation, the culture medium was aspirated, and formazan crystals were dissolved in DMSO. Absorbance was measured at 570 nm with a reference wavelength of 620 nm using a Multiskan RC microplate reader (LabSystems, Vantaa, Finland). Cell viability was calculated as a percentage relative to untreated control cells. Anti-proliferative effect was determined as growth inhibition (%) = 100 − cell viability (%).

### 2.4. Scratch Assay

Cell migration was assessed using a scratch wound healing assay. CT-26 cells were seeded at a density of 1.4 × 10^6^ cells per well in 6-well plates in serum-free DMEM. Upon reaching 80% confluence, cells were transfected with individual oligonucleotides or triple combination at a total concentration of 120 nM (40 nM of each oligonucleotide in combination) as described above. At 24 h post-transfection, three parallel scratch wounds were created in each well using a sterile micropipette tip. Detached cells were removed by washing twice with sterile phosphate-buffered saline (PBS), and fresh DMEM containing 10% FBS and 1% antibiotic–antimycotic solution was added. Wound closure was monitored by capturing phase-contrast microscopy images (Zeiss Primo Vert, Carl Zeiss, Jena, Germany) at 0 and 48 h post-scratching. For each scratch, at least five images along its length were acquired. Scratch width was measured using ImageJ software v 1.54j (National Institutes of Health, Bethesda, MD, USA) and calculated as the mean ± SEM of at least five measurements per scratch. The average scratch width per well at each time point was determined from three independent scratches. Cell migration rate was calculated using the following formula: migration rate (%) = (1 − W_48_/W_0_) × 100%, where W_48_ and W_0_ represent scratch width at 48 h and 0 h, respectively.

### 2.5. Stem-Loop PCR

Total RNA was isolated from CT-26 cells 48 h post-transfection with oligonucleotides using RIzol Reagent (DIAM, Moscow, Russia) according to the manufacturer’s protocol. Reverse transcription was carried out in a 20 µL reaction mixture containing RT buffer (50 mM Tris–HCl pH 8.3, 50 mM KCl, 4 mM MgCl_2_, 0.5 mM dNTP, 10 mM DTT), 100 U M-MuLV-RH reverse transcriptase (Biolabmix, Novosibirsk, Russia), 3 µg total RNA, and 50 nM specific RT primers (Table S1). The reaction proceeded under the following conditions: 16 °C for 30 min (1 cycle), followed by 40 cycles of 30 °C for 30 s and 42 °C for 30 s. PCR amplification was performed using BioMaster HS-qPCR SYBR Blue mix (Biolabmix, Novosibirsk, Russia) on a Bio-Rad iQ5 Real-Time PCR Detection System (Bio-Rad Laboratories, Hercules, CA, USA) according to the manufacturer’s protocol. Briefly, the PCR reaction contained 20 µL of reaction mixture comprising PCR buffer (50 mM Tris–HCl pH 8.5, 50 mM KCl, 1.5 mM MgCl_2_, 0.2 mM each dNTP, 0.03 U Taq polymerase, 0.0125% Tween-20, SYBR Green I), 5 µL cDNA sample (1:10 dilution), and 0.25 µM specific PCR primers (Table S1). Thermal cycling conditions included initial denaturation at 95 °C for 4 min, followed by 40 cycles of 95 °C for 40 s, 60 °C for 30 s, and 72 °C for 30 s. Relative miRNA expression levels were determined using the ΔΔCt method with U6 as the reference gene for in vitro experiments and U6 and SNORD43 as dual reference genes for in vivo tumor tissue samples.

### 2.6. Mice

Ten- to twelve-week-old BALB/c female mice were obtained from the vivarium of Institute of Chemical Biology and Fundamental Medicine SB RAS (Novosibirsk, Russia). Mice were housed in plastic cages in standard daylight conditions (12/12 h light/dark cycle). Water and food were supplied ad libitum. All animal procedures were conducted in strict compliance with the guidelines for the proper use and care of laboratory animals (ECC Directive 2010/63/EU) and ARRIVE guidelines 2.0 [40,41]. The experimental protocols were approved by the Committee on the Ethics of Animal Experiments of the Institute of Cytology and Genetics SB RAS (ethical approval number 217 from 25 February 2025).

### 2.7. Preparation of Oligonucleotide–Liposome Complexes for In Vivo Experiments

Oligonucleotide delivery was performed using cationic liposomes 2X3-DOPE or folate-containing liposomes F13 [38,42] at a stock concentration of 1 mM. Lipoplexes were prepared prior to administration by mixing equal volumes of oligonucleotide and liposome solutions in IMDM at N/P ratios of 2/1 or 4/1, followed by 20 min incubation at room temperature. For monotherapy, 10 μg of individual oligonucleotide in 200 µL was mixed with 200 µL of liposome solution. For triple combination therapy, three separate complexes were prepared (3.3 µg of each oligonucleotide in 67 µL mixed with 67 µL of liposome solution) and then combined, yielding a final injection volume of 400 µL in both cases.

### 2.8. CT-26 Tumor Models

To identify the optimal experimental model, CT-26 tumor development was evaluated following subcutaneous, intramuscular, intraperitoneal, and intravenous administration routes. For subcutaneous and intramuscular models, CT-26 cells (1.5 × 10^5^ cells in 0.1 mL IMDM) were injected into the left flank or left femoral muscle, respectively. For intraperitoneal and intravenous models, CT-26 cells (1.5 × 10^5^ cells in 0.4 mL IMDM) were inoculated intraperitoneally or injected intravenously via the tail vein, respectively. Detailed characterization of intraperitoneal adenomatosis progression was performed following intraperitoneal inoculation of CT-26 cells (1 × 10^5^ cells in 0.4 mL IMDM). Mice were euthanized on days 10, 12, 14, 16, and 18, and the digestive tract with adenomas, spleen, thymus, liver, and kidneys were collected. Organ indices were calculated using the following formula: organ index (%) = (organ weight/body weight) × 100. For immunosuppression analysis, half of the spleen was placed in ice-cold PBS, and blood was collected from the orbital sinus into EDTA-coated tubes.

### 2.9. In Vivo Biodistribution Studies

Biodistribution of Cy5.5-labeled μ-21 oligonucleotide was assessed using subcutaneous and intraperitoneal CT-26 tumor models induced as described above. At day 14 post-tumor inoculation, mice received either peritumoral injections (0.1 mL, subcutaneous model) or intraperitoneal injections (0.4 mL, intraperitoneal model) containing 10 μg of Cy5.5-μ-21 precomplexed with F13 or 2X3-DOPE liposomes at N/P ratios of 2/1 or 4/1. At 24 h post-injection, mice were euthanized under isoflurane anesthesia, and organs (lungs, heart, liver, spleen, kidneys, digestive tract, and tumors) were isolated for ex vivo imaging. Near-infrared fluorescence (Cy5.5: excitation 620 nm, emission 700 nm) was detected using an IVIS Lumina X5 Imaging System (Revvity, Waltham, MA, USA). Fluorescence intensity was quantified using Living Image software v 4.5.5 (Revvity, Waltham, MA, USA). For each sample, an ROI was defined using an automated threshold set at 17% of maximum pixel intensity. Fluorescence signal was measured as average radiant efficiency ([p/s/cm^2^/sr]/[μW/cm^2^]) and normalized to tissue area. Images were exported as 16-bit TIFF files.

### 2.10. Anti-Tumor Studies

To assess anti-tumor effect, the CT-26 peritoneal adenomatosis model was established by intraperitoneal inoculation of 1 × 10^5^ cells in 0.4 mL IMDM. On day 3 post-inoculation, mice were randomized into six groups (*n* = 16 per group): (1) untreated control receiving IMDM; (2) negative control receiving control oligonucleotide μ-Scr (10 μg/mouse); (3)–(5) monotherapy groups receiving oligonucleotides μ-21, μ-155, or μ-17 (10 μg/mouse each); and (6) combination therapy group (Combi) receiving a tri-component formulation (3.3 μg of each oligonucleotide per mouse). Treatment consisted of eight intraperitoneal injections of oligonucleotide-liposome complexes (N/P 2/1, 0.4 mL IMDM), administered at 4-day intervals on days 3, 7, 11, 15, 19, 23, 27, and 31. To assess the dynamics of therapeutic response, four mice per group were euthanized on days 14 and 18 for therapeutic efficacy assessment. Isolated organs (digestive tract with adenomas, spleen, thymus, liver, and kidneys) were weighed, and organ indices were calculated as described above. Thymus and intestinal tissues with adenomas were fixed in 10% neutral buffered formalin (BioVitrum, Moscow, Russia) for subsequent histological and morphometric analysis. Spleens and blood were collected and processed for immunosuppression analysis as described below. Adenoma samples from each treatment group were collected in RIzol Reagent (DIAM, Moscow, Russia) and homogenized using Lysing Matrix D (MP Biomedicals, Santa Ana, CA, USA) and a FastPrep-24 5G homogenizer (MP Biomedicals, Santa Ana, CA, USA), followed by total RNA isolation and stem-loop RT-PCR as described above. The remaining mice (n = 8 per group) were monitored for survival analysis. Survival data were analyzed using Kaplan–Meier curves and mean survival times. The increase in lifespan (ILS) was calculated as: ILS (%) = [(mean survival time of treated group − mean survival time of control group)/mean survival time of control group] × 100.

### 2.11. Histology

Formalin-fixed thymus and adenomas were dehydrated in ascending ethanol concentrations and embedded in HISTOMIX paraffin (BioVitrum, Moscow, Russia). Paraffin sections (up to 5 μm) were sliced on a Microm HM 355S microtome (Thermo Fisher Scientific, Waltham, MA, USA) and stained with hematoxylin and eosin. Images were examined and scanned using Axiostar Plus microscope equipped with Axiocam MRc5 digital camera (Zeiss, Oberkochen, Germany) at magnifications of ×100 and ×400.

For immunohistochemical analysis, adenoma sections were deparaffinized and rehydrated. Antigen retrieval was performed using microwave treatment at 700 W. Sections were incubated with anti-CD3 primary antibodies (ab182981, Abcam, Boston, MA, USA) according to the manufacturer’s protocol, followed by incubation with horseradish peroxidase (HRP)-conjugated secondary antibodies. Immunostaining was visualized using 3,3′-diaminobenzidine (DAB) substrate (Rabbit Specific HRP/DAB (ABC) Detection IHC Kit, ab64261, Abcam, Boston, MA, USA) and counterstained with Mayer’s hematoxylin.

Morphometric analysis of tumor sections was performed using a closed test system with a testing area equal to 3.2 × 10^6^ μm^2^ and included evaluation of the numerical densities (Nv) of mitoses and CD3^+^ cells outside the necrotic destruction foci, indicating the number of mitotic cells in the square unit, 3.2 × 10^6^ μm^2^ in this case. For each parameter, ten random fields were studied in each specimen forming 40 random fields for each group of mice.

Tumor volumes were calculated using scans of stained tumor sections as follows: V = (D × d^2^)/2, where D is the longest diameter of the tumor node and d is the shortest diameter of the tumor node perpendicular to D.

### 2.12. Peripheral Blood Collection

Peripheral blood was collected from the retro-orbital sinus of the mice using EDTA-coated tubes to prevent blood coagulation. Red blood cells were lysed with RBC lysis buffer (0.15 M NH_4_Cl, 10 mM NaHCO_3_, and 0.1 mM EDTA; Sigma-Aldrich, Darmstadt, Germany) for 5 min at room temperature, followed by washing with a large amount of PBS buffer. The viability of isolated blood cells was measured by trypan blue exclusion assay. Blood cells were resuspended in staining buffer (PBS with 2% FBS) for subsequent flow cytometry analysis.

### 2.13. Splenocyte Isolation

Excised spleens were placed in a sterile Petri dish containing 5 mL PBS and gently homogenized using the sterile syringe plunger. The cell suspension was passed through a 70 μm cell strainer (Corning, Glendale, AZ, USA) to obtain a single-cell suspension. Red blood cells were lysed with RBC lysis buffer. Cell viability was evaluated by trypan blue exclusion assay. Splenocytes were resuspended in staining buffer (PBS with 2% FBS) for subsequent flow cytometry analysis.

### 2.14. Flow Cytometry Analysis

The phenotype of immune cells was evaluated by assessing the expression of extracellular markers using flow cytometry. A total of 0.5 × 10^6^ of peripheral blood cells or splenocytes in 100 µL of staining buffer (PBS with 2% FBS) were used in each test. First, cells were Fc-blocked with anti-mouse CD16/CD32 IgG antibodies (BD Biosciences, San Diego, CA, USA) according to the manufacturer’s recommendations. To characterize the immune cells, the following anti-mouse monoclonal antibodies were used: monoclonal anti-CD45-FITC (BD Biosciences, San Diego, CA, USA), CD11b-PerCP (Elabscience, Houston, TX, USA), Ly6C-BV605 (Sony, San Jose, CA, USA), and Ly6G-PE (Elabscience, Houston, TX, USA). The cells were stained with antibodies (1:100) for 30 min at room temperature, washed twice with staining buffer, and then fixed in 2% formaldehyde in PBS. Flow cytometry measurements were performed using a NovoCyte 3000 flow cytometer (ACEA Biosciences, San Diego, CA, USA), and at least 10,000 events were acquired for each sample. The data were processed using NovoExpress software v. 1.1.0 (ACEA Biosciences, San Diego, CA, USA). Gating strategies for MDSCs are presented in Figure S1.

### 2.15. ELISA

Mouse peripheral blood was collected into test tubes without anti-coagulant, incubated at 37 °C for 30 min to allow clotting with subsequent incubation at 4 °C overnight. The samples were centrifuged at 4000 rpm for 20 min at 4 °C. The resulting serum was carefully transferred into new test tubes and centrifuged a second time (4000 rpm, 4 °C, 20 min) to remove residual cellular debris. The serum was collected into fresh tubes and stored at −80 °C until analysis.

Serum levels of IFN-γ, IL-6, and TNF-α were analyzed using mouse-specific ELISA kits (Thermo Fisher Scientific, Waltham, MA, USA) according to the manufacturer’s instructions. Absorbance was measured at 450 nm using a Multiskan RC microplate reader (Thermo LabSystems, Helsinki, Finland).

### 2.16. Western Blot Analysis

On day 18, adenoma specimens were lysed in RIPA buffer (Thermo Fisher Scientific, Waltham, MA, USA) and homogenized using Lysing Matrix D with a FastPrep-24 5G homogenizer (MP Biomedicals, Santa Ana, CA, USA). Protein samples (30 µg) were separated by 12.5% SDS-PAGE and transferred to PVDF membranes (Immobilon-P, Burlington, MA, USA) using a Criterion blotter (Bio-Rad Laboratories, Hercules, CA, USA). Membranes were blocked in 5% non-fat milk in TBST and incubated overnight at 4 °C with primary antibodies: rabbit polyclonal anti-E2F1 and anti-SOCS1 (1:400 and 1:800, respectively; ARG59557 and ARG55607, Arigo Biolaboratories, Hsinchu City, Taiwan), rabbit monoclonal anti-PDCD4 (1:800; ab79405, Abcam, UK), and anti-GAPDH (1:10,000; A19056, ABclonal, Wuhan, Hubei, China). Following TBST washes, membranes were incubated with HRP-conjugated goat anti-rabbit secondary antibodies (1:3000; Cloud-Clone Corp., Wuhan, Hubei, China) for 1 h. Protein bands were visualized using an ECL kit (Elabscience, Wuhan, Hubei, China) and the iBright 1500 system (Invitrogen, Carlsbad, CA, USA). Band intensities were quantified using Gel Analyzer v.23.1.1 software and normalized to GAPDH.

### 2.17. Enzymatic Dissociation of Tumor Tissue

Freshly excised tumor tissue (0.15 g) was washed twice in PBS and cut into small fragments in 1 mL serum-free medium, followed by the addition of 2 mg/mL collagenase type I (Gibco, Thermo Fisher Scientific, Waltham, MA, USA) and 0.03 mg/mL Dispase (Gibco, Thermo Fisher Scientific, Waltham, MA, USA) and incubation at 37 °C for 3 h on a shaker (rotation at 300 rpm), with gentle inversion or pipetting every 30 min. After incubation, the suspension was passed through a 70 µm cell strainer and washed in a medium containing 2% FBS. Cell viability was evaluated by trypan blue exclusion assay, and cells were resuspended in staining buffer (PBS with 2% FBS) for subsequent flow cytometry analysis.

### 2.18. Statistical Analyses

For normally distributed data, statistical comparisons were conducted using a two-tailed unpaired Student’s *t*-test or one-way ANOVA followed by Tukey’s multiple comparisons test. For data that did not meet normality assumptions, the Mann–Whitney U test was employed. Survival analysis was performed using Kaplan–Meier curves with the log-rank (Mantel–Cox) test. A *p*-value < 0.05 was considered statistically significant. Data are presented as mean ± SEM unless otherwise indicated. Statistical analysis was performed using GraphPad Prism version 8.4.3 (GraphPad Software, San Diego, CA, USA).

## 3. Results

### 3.1. Functional and Molecular Responses of CT-26 Cells to miRNA-Targeted Oligonucleotide Treatment

The anti-tumor potential of miRNA-targeted oligonucleotides was investigated using colorectal carcinoma CT-26 cell culture and a syngeneic mouse model, characterized by aggressive growth. Fully modified methylsulfonyl phosphoramidate deoxyoligonucleotides (mesyl- or µ-ONs) targeting oncogenic miR-21, miR-155, and miR-17 (µ-21, µ-155, and µ-17, respectively) were used as therapeutic agents (Figure 1a).

To initially assess the therapeutic potential of miR-21, miR-17, and miR-155 silencing, we investigated the effect of specific oligonucleotides on key oncogenic properties of CT-26 colorectal cancer cells in vitro, particularly on their proliferative and migratory activities. Cationic 2X3-DOPE liposomes were used as a delivery vehicle [38].

Cell viability analysis (MTT assay) showed that at 72 h post-transfection, the μ-21 oligonucleotide showed the strongest anti-proliferative effect, achieving 50% growth inhibition at 85 nM (Figure 1b). The μ-17 oligonucleotide required 120 nM to reach 50% efficacy, while the μ-155 oligonucleotide demonstrated maximal inhibition of 30% at 80 nM with no further enhancement at higher concentrations (Figure 1b). To assess the efficacy of combination treatment, we selected a concentration of 40 nM for each oligonucleotide, as this dose provides substantial efficacy approaching maximal effect within the linear response phase. At this concentration, the anti-proliferative effects reached 21%, 27%, and 37% for μ-155, μ-17, and μ-21, respectively (Figure 1b). The triple combination of µ-ONs (Combi) was compared with individual oligonucleotides at an equivalent total concentration of 120 nM. The results demonstrated that Combi induced a 40% decrease in cell viability, which approximated the effect of μ-17 and μ-21 at equivalent concentrations, though it did not surpass the effect of individual oligonucleotides used at the cumulative 120 nM dose (Figure 1c).

Migration assays demonstrated that individual oligonucleotides reduced cell motility by approximately 35%. However, Combi produced a rather weak, statistically non-significant anti-migratory effect (Figure 1d).

Analysis of miRNA levels confirmed specific knockdown of each miRNA target by approximately 65% following individual oligonucleotide treatment, while the application of Combi maintained efficient suppression and even enhanced the inhibitory effect, particularly for miR-21 and miR-17 (Figure 1e).

These findings demonstrate that individual oligonucleotides targeting miR-21, miR-17, and miR-155 exhibited considerable therapeutic potential with significant anti-proliferative and anti-migratory effects. Similarly, the combination approach effectively suppressed target miRNAs and produced substantial anti-proliferative effects, while the non-significant anti-migratory response may result from the lower individual concentrations of µ-ONs used in the combinatorial treatment.

### 3.2. Characterization of CT-26 Intraperitoneal Tumor Model: Kinetics of Neoplastic Progression

To select the optimal conditions for CT-26 tumor growth, the parameters of tumor development induced by various administration routes of CT-26 cells in BALB/c mice were analyzed: subcutaneous, intramuscular, intraperitoneal, and intravenous. Tumor progression analysis revealed that intraperitoneal injection induced the formation of multiple adenomas distributed along the intestinal mesentery, while intravenous injection resulted in tumor nodule development in pulmonary tissues. Subcutaneous and intramuscular inoculation generated rapidly growing tumors at injection sites with frequent cases of ulcerative necrosis. Necrotic foci formation in subcutaneous and intramuscular tumors substantially compromises accurate tumor volume assessment, hinders comprehensive histological analysis, and may substantially reduce therapeutic agent penetration into tumor tissues. In contrast, the intraperitoneal tumor model was characterized by the formation of multiple non-necrotic adenomas, providing more favorable conditions for reliable assessment of treatment efficacy. Therefore, subsequent studies focused on the CT-26 intraperitoneal adenomatosis model.

**Figure 2:**
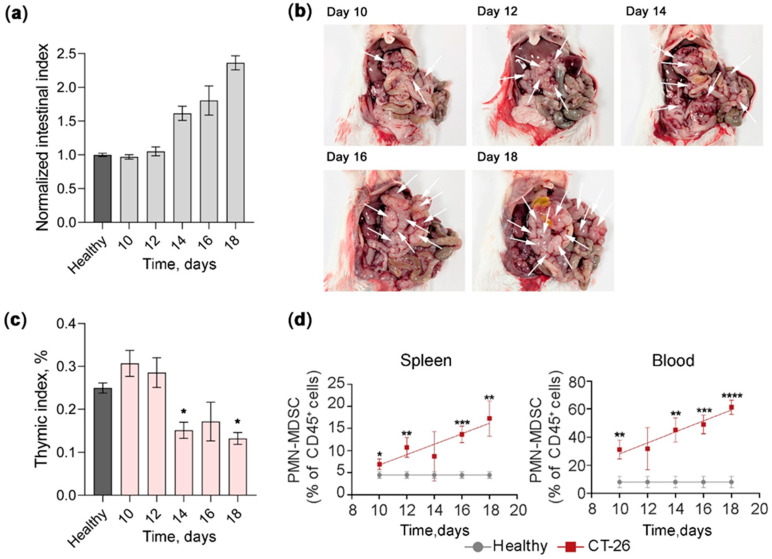
Characterization of intraperitoneal CT-26 colorectal carcinoma. (**a**) Normalized intestinal index in mice with progressive CT-26 intraperitoneal adenomatosis. CT-26 cells were intraperitoneally implanted into BALB/c mice (10^5^ cells/mouse, *n* = 6). (**b**) Representative photographs of intraperitoneal adenoma development showing tumor progression. Arrows indicate adenoma lesions. (**c**) Dynamics of thymic index in tumor-bearing mice. The data were analyzed using Mann–Whitney U test. (**d**) Percentage of myeloid-derived suppressor cells (MDSC) CD11b^+^Ly6G^+^Ly6C^low^ PMN-MDSCs in the spleen and peripheral blood during tumor progression. Data are shown for healthy animals (grey line) and CT-26 tumor-bearing mice (red line). The data were analyzed using one-way ANOVA followed by Tukey’s post hoc test. Data represent mean ± SEM. *, **, ***, ****—significant differences from the healthy control with *p* < 0.05, *p* < 0.01, *p* < 0.001, and *p* < 0.0001, respectively.

Detailed analysis of the dynamics of intraperitoneal tumor progression (Figure 2a,b) revealed the emergence of discrete multiple adenomas within the mesenteric tissue by day 10 after tumor cell inoculation. Tumor development was assessed by calculating the intestinal indices normalized to this parameter in healthy animals (control) (Figure 2a). Progressive adenoma growth exhibited a typical biphasic progression pattern with an initial lag phase followed by rapid exponential expansion. This acceleration became markedly evident between days 12 and 14, characterized by a noticeable increase in the intestinal index (Figure 2a), which reached a two-fold elevation by day 18 compared to healthy animals. Starting from day 14, tumor progression was accompanied by pronounced thymic atrophy, evidenced by a two-fold decrease in the thymic indices (Figure 2c), revealing the inhibiting impact of CT-26 on the immune system.

Investigation of the immunosuppressive status in mice with developing peritoneal adenomatosis revealed a progressive accumulation of myeloid-derived suppressor cells (MDSCs), specifically the polymorphonuclear subset (PMN-MDSCs), in the spleen and peripheral blood of tumor-bearing animals. Tumor progression was associated with a significant, continuous increase in PMN-MDSCs, which was most pronounced in the peripheral blood. In the spleen, the PMN-MDSC population increased from 5% in healthy controls to 7% in tumor-bearing mice by day 10, escalating markedly to 17% by day 18 of tumor progression (Figure 2d, left panel). More strikingly, the level of PMN-MDSCs in peripheral blood increased from 8% in control animals to 32% in tumor-bearing mice by day 10, followed by a remarkable progressive accumulation reaching 60% by day 18 (Figure 2d, right panel). At the same time, monocytic MDSCs (M-MDSCs) comprised less than 4% of CD45^+^ cells in both spleen and peripheral blood while still maintaining a correlation between their frequency and tumor progression (Supplementary Figure S2). The level of tumor infiltration by both PMN-MDSCs and M-MDSCs was low (<0.09% of total cells) (Supplementary Figure S3). Together with thymic index measurements, these results indicate that the intraperitoneal CT-26 adenomatosis model exhibits pronounced systemic immunosuppression, driven by progressive MDSC accumulation, closely recapitulating the immunosuppressive microenvironment of human colorectal cancer and peritoneal malignancies [43,44,45].

**Figure 3:**
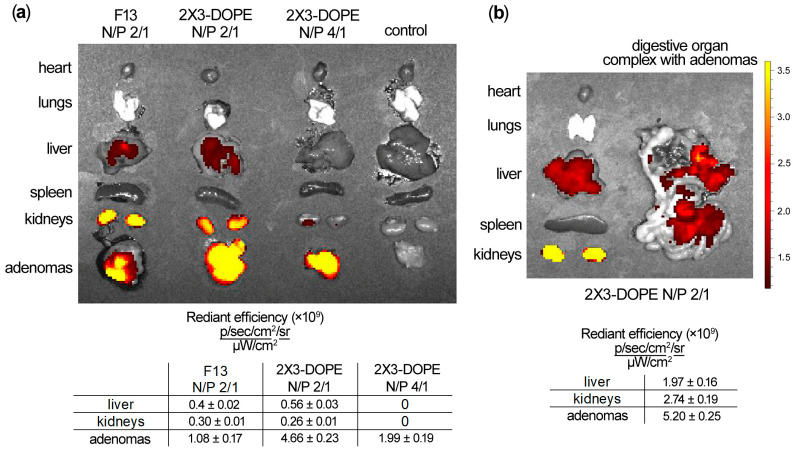
Biodistribution of Cy5.5-labeled oligonucleotide in CT-26 tumor-bearing mice following different administration routes. (**a**) Peritumoral administration of Cy5.5-labeled oligonucleotide (10 µg/mouse) precomplexed with cationic liposomes F13 (N/P ratio 2/1) or 2X3-DOPE (N/P ratios 2/1 and 4/1) in the subcutaneous CT-26 tumor model. (**b**) Intraperitoneal administration of Cy5.5-labeled µ-ON precomplexed with cationic liposomes 2X3-DOPE (N/P ratios 2/1) in mice with induced CT-26 adenomatosis. Real-time fluorescent images of dissected organs were taken 24 h after oligonucleotide injection. Control—non-injected mouse.

To select the optimal delivery system and conditions for miRNA-targeted oligonucleotide transport to tumor tissue, the accumulation of Cy5.5-labeled oligonucleotide was analyzed using in vivo imaging in both subcutaneous tumor nodules and intraperitoneal adenomas generated by CT-26 cell inoculation. Comparative biodistribution studies revealed that 24 h following peritumoral injection, Cy5.5-labeled oligonucleotide formulated with 2X3-DOPE liposomes demonstrated superior tumor penetration and retention compared to the folate-equipped F13 liposomal formulation at equivalent 2/1 N/P ratios (Figure 3a). An increase in the N/P ratio to 4/1 failed to enhance intratumoral oligonucleotide accumulation (Figure 3a). Subsequently, 2X3-DOPE liposomes were employed to assess the accumulation of Cy5.5-labeled mesyl oligonucleotides within the intraperitoneal adenomatosis model. Intraperitoneal administration of fluorescent oligonucleotide precomplexed with 2X3-DOPE liposomes (N/P 2/1) promoted efficient and homogeneous accumulation of µ-ONs directly in mesenteric tumor nodules 24 h post-administration (Figure 3b).

Thus, the peritoneal adenomatosis model represents an optimal experimental system due to the formation of small, non-necrotic intraperitoneal adenomas distributed along the intestinal mesentery, combined with pronounced systemic immunosuppression. Despite the limitation that tumor growth monitoring requires endpoint analysis, this model offers the critical advantages of uniform and efficient drug delivery to tumor tissues and reliable evaluation of the therapeutic efficacy of miRNA-targeted oligonucleotides.

### 3.3. Therapeutic Effects of miRNA-Targeted Oligonucleotides on CT-26 Tumor Progression

**Figure 4:**
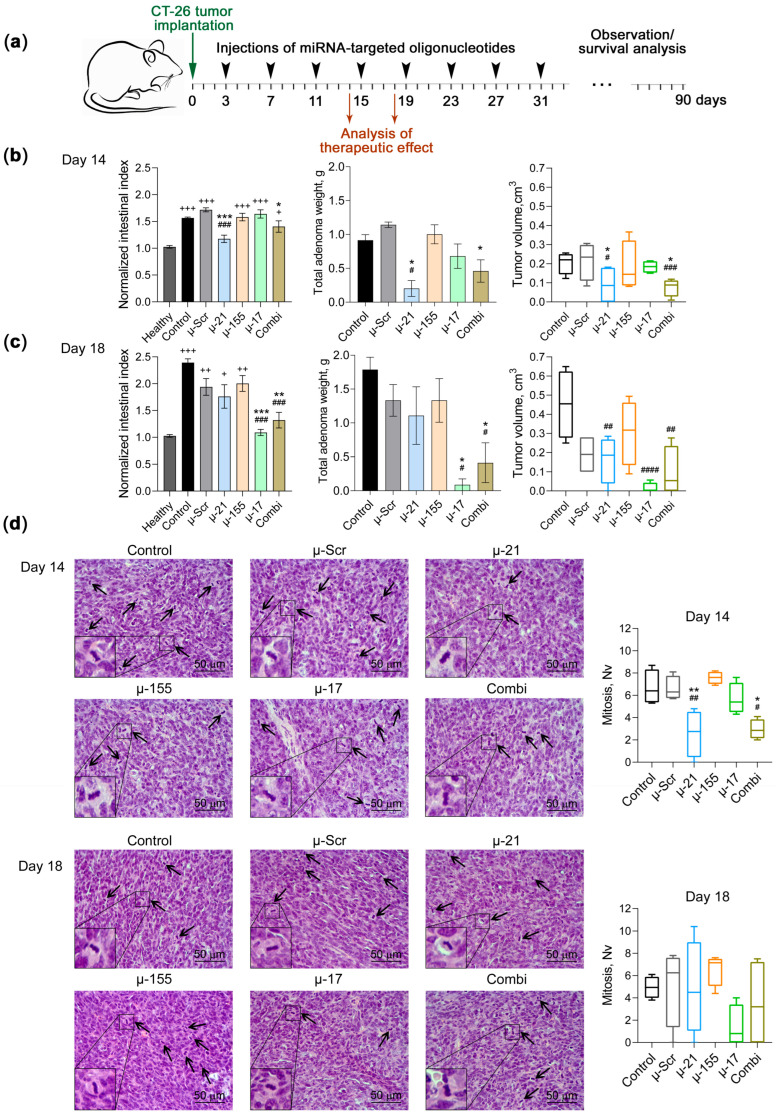
Anti-tumor efficacy of miR-21, miR-17, and miR-155-targeted therapy in the CT-26 peritoneal adenomatosis model. (**a**) Experimental scheme showing the regimen of intraperitoneal administration of µ-21, µ-155, and µ-17 oligonucleotides and their combination (initial number of mice in each group *n* = 16). Black arrows indicate days of µ-ON administration. (**b**,**c**) Normalized intestinal index, total adenoma weight and total tumor volume in CT-26 tumor-bearing mice on days 14 and 18 of CT-26 peritoneal adenomatosis progression, respectively (*n* = 8 for each group with *n* = 4 for each time point). (**d**) Representative images of hematoxylin and eosin-stained tumor tissue sections and morphometric quantification of mitotic activity. Magnification ×400. Arrows indicate mitotic events. Representative mitoses at higher magnification are shown in the lower left corner. Data represent mean ± SEM. The data were analyzed using Mann–Whitney U test. ^#^, ^##^, ^###^, ^####^—significant differences from control with *p* < 0.05, *p* < 0.01, *p* < 0.001, and *p* < 0.0001, respectively. *, **, ***—significant differences from μ-Scr with *p* < 0.05, *p* < 0.01, and *p* < 0.001, respectively. ^+^, ^++^, ^+++^—significant differences from healthy animals with *p* < 0.05, *p* < 0.01, and *p* < 0.001, respectively.

The anti-tumor potential of oligonucleotide-mediated therapy targeting miR-21, miR-155, and miR-17 was evaluated using the CT-26 peritoneal adenomatosis model. Tumor development was initiated on day 0 by intraperitoneal implantation of CT-26 cells (1 × 10^5^ cells in 0.4 mL IMDM) into BALB/c mice. Treatment was initiated on day 3 after CT-26 implantation, and the therapeutic protocol included regular intraperitoneal administration of µ-ONs pre-complexed with 2X3-DOPE liposomes (2/1 N/P ratio) every 4 days until day 31 (Figure 4a). Six groups were included in the study: (1) control group receiving IMDM injections; (2) negative control group receiving µ-Scr oligonucleotide (10 μg/mouse); (3)–(5) monotherapy groups receiving individual oligonucleotides µ-21, µ-17, or µ-155, respectively (10 μg/mouse); and (6) Combi group receiving a tri-component oligonucleotide formulation (3.3 μg each, 10 μg total/mouse). The standard dose range for cationic liposome-mediated oligonucleotide delivery in murine tumor models is approximately 5–50 µg per injection [46,47]. We employed 10 µg per injection based on the enhanced nuclease resistance of mesyl oligonucleotides and demonstrated effective target inhibition without observable toxicity in our previous work [11,38,48]. During the study, mice were monitored for up to 90 days. Therapeutic efficacy was assessed on days 14 and 18 of tumor progression, corresponding to 72 h after the third and fourth oligonucleotide injections, respectively. Assessment included evaluation of organ indices, measurement of total adenoma volume, histological analysis of tumors and thymus, and assessment of the effects of therapy on the expansion of immunosuppressive PMN-MDSCs in mice.

The anti-tumor effect was evaluated based on changes in the intestinal indices normalized to healthy mice, mean total adenoma weight and volume in control and experimental groups. Analysis of therapeutic efficacy revealed that treatment with miRNA-targeted µ-ONs exhibited varying dynamics and degree of anti-tumor activity compared to untreated control and animals receiving μ-Scr (Figure 4b,c and Figure S4). μ-155 demonstrated minimal therapeutic effect throughout the experimental period, with intestinal index as well as tumor weight and volume remaining comparable to control groups on days 14 and 18, indicating limited anti-tumor activity under the current treatment regimen. μ-21 exhibited a therapeutic profile characterized by marked early efficacy followed by gradual attenuation of the response. On day 14, μ-21 treatment achieved substantial anti-tumor effects, with the intestinal index only marginally exceeding values observed in healthy animals, total adenoma weight decreased by approximately 4-fold, and mean adenoma volume decreased approximately two-fold compared to controls (Figure 4b). By day 18, therapeutic activity of µ-21 declined, with the intestinal index approaching control values, while tumor volume suppression persisted, remaining significantly reduced relative to the control group (Figure 4c). This partial loss of therapeutic efficacy suggests either compensatory tumor adaptation or potential development of resistance mechanisms. In contrast, μ-17 demonstrated a delayed but highly potent anti-tumor response. While showing minimal efficacy on day 14, μ-17 treatment exhibited marked therapeutic activity by day 18, with an approximately two-fold reduction in intestinal index and 10-fold decrease in tumor weight and volume compared to the control group, indicating time-dependent mechanisms of action requiring extended treatment duration for optimal efficacy (Figure 4c). Combi therapy displayed consistent anti-tumor activity across both time points. On day 14, treatment with the tri-component formulation resulted in reduced intestinal index and tumor weight and volume compared to controls. This effect persisted through day 18, maintaining the intestinal index at approximately two-fold lower levels and substantial suppression of adenoma growth relative to the control group (Figure 4c). This stable efficacy profile suggests that the multi-target oligonucleotide strategy effectively combines individual therapeutic advantages to achieve durable anti-tumor responses.

Histologically, CT-26 adenomas were represented by well-formed tumor nodes with densely packed neoplastic cells (Figure 4d). Scattered areas of necrosis, moderately infiltrated by neutrophils with an admixture of lymphocytes and macrophages and in some cases occupying the entire adenomatous nodule, were observed within the tumor tissue. Evaluation of the stromal component of CT-26 revealed the enrichment of the tumor with immature blood vessels with thin walls as well as pronounced congestion of local capillaries with areas of hemorrhages. Tumors in the control and µ-Scr-treated groups were characterized by high mitotic activity with a numerical density of cells in the state of mitosis amounted of approximately six and five per square unit on days 14 and 18 of tumor growth, respectively (Figure 4d). Administration of µ-155 did not have any positive effect on mitoses, making them no different from controls throughout the observation period. Treatment with µ-21 led to a 2.6-fold decrease in the number of mitoses in the tumor tissue compared to controls on day 14 of tumor growth and did not affect this parameter on day 18 (Figure 4d). Conversely, µ-17 administration did not affect mitoses on day 14 but effectively reduced the number of mitoses by 3.6-fold on day 18 of tumor growth compared to controls (Figure 4d). Combi was effective across both time points, demonstrating a 2.3- and 1.4-fold reduction in the numerical density of mitoses on days 14 and 18 of tumor growth, respectively, compared to controls (Figure 4d). The presented results on the mitotic activity of the CT-26 tumors correlate well with the data on the anti-tumor effect of the applied oligonucleotide treatment.

Evaluation of miRNA levels in adenoma tissues on day 18 (three days after the fourth injection) confirmed miRNA-target suppression in treatment groups (Figure S5a). Oligonucleotide monotherapy at a dose of 10 μg/mouse achieved 30–65% reduction in target miRNA expression, demonstrating significant silencing activity under the applied treatment regimen. Combi therapy demonstrated 30% suppression of all three targets despite a three-fold dose reduction of each oligonucleotide (3.3 μg). The miRNA-specific effects of µ-ONs were additionally validated at the protein level in adenoma tissue. On day 18, the protein level of the direct miR-21 target PDCD4 was increased by 3.8- and five-fold following µ-21 and Combi treatment, respectively (Figure S5b). The expression of the direct miR-155 target SOCS1 was increased by 1.3-fold after Combi treatment, while the protein level of the direct miR-17 target E2F1 was elevated by two- and five-fold after µ-17 and Combi treatment, respectively, compared to control (Figure S5b).

### 3.4. Systemic Immune Effects of miRNA-Targeted Oligonucleotides in the CT-26 Model

The thymus plays a crucial role in anti-tumor immunity through generation of naive T lymphocytes and maintenance of peripheral immune surveillance. However, tumor progression frequently induces thymic atrophy due to systemic immunosuppression and chronic inflammation, which compromises the immune response [49,50].

**Figure 5:**
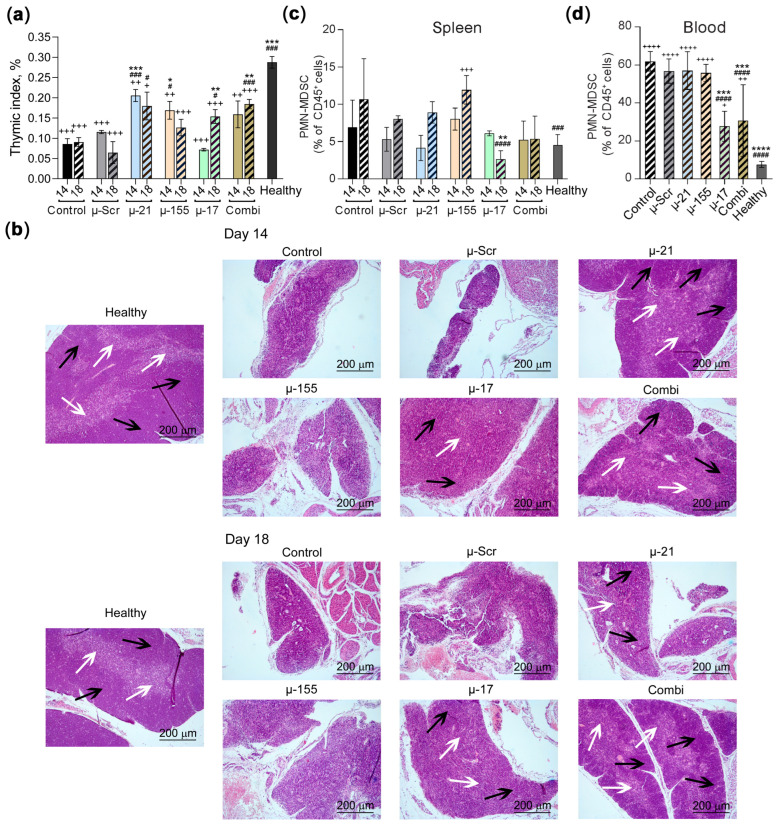
Immunomodulatory effects of miR-21, miR-17, and miR-155-targeted therapy on thymus and suppressor cell populations in the CT-26 colorectal cancer model. (**a**) Thymic index in tumor-bearing mice on days 14 (solid bars) and 18 (striped bars) (*n* = 8 for each group with *n* = 4 for each time point). The data were analyzed using the Mann–Whitney U test. (**b**) Representative histological sections of the thymus from experimental animals showing typical morphology in each group. Hematoxylin and eosin staining. Magification ×100. Black arrows indicate the thymic cortex; white arrows indicate the thymic medulla. (**c**) Percentage of PMN-MDSCs in spleen on days 14 (solid bars) and 18 (striped bars). (**d**) Percentage of PMN-MDSC in peripheral blood of mice on day 18. Data represent mean ± SEM. The data were analyzed using one-way ANOVA with Tukey’s post hoc test. ^#^, ^###^, ^####^—significant differences from control with *p* < 0.05, *p* < 0.001, and *p* < 0.0001, respectively. *, **, ***, ****—significant differences from μ-Scr with *p* < 0.05, *p* < 0.01, *p* < 0.001, and *p* < 0.0001, respectively. ^+^, ^++^, ^+++^, ^++++^—significant differences from healthy animals with *p* < 0.05, *p* < 0.01, *p* < 0.001 and *p* < 0.0001, respectively.

Assessment of the thymic index revealed that tumor development induced profound organ involution in experimental animals (Figure 5a,b). Histological analysis demonstrated that the thymus of CT-26-bearing control animals exhibited severe atrophy characterized by nearly complete depletion of lymphoid elements and extensive replacement of thymic parenchyma with adipose and fibrotic tissues. In residual thymic tissue, cortical-medullary architectural inversion was observed. µ-Scr did not have any effect on the thymus structure. Furthermore, at the later observation time point (day 18 versus day 14 of tumor progression), complete thymic collapse was detected in the control group (Figure 5b). Treatment with μ-155, despite demonstrating minimal anti-tumor activity, prevented early-stage organ atrophy, as evidenced by elevated thymic index values compared to control groups at day 14. However, histological examination revealed that μ-155 administration failed to preserve normal thymic architecture at either time point, with structural deterioration comparable to controls (Figure 5a,b). μ-17 treatment did not significantly affect thymic dimensions on day 14. However, by day 18, along with pronounced anti-tumor efficacy, a notable positive effect on thymus function was observed, manifested by an increased thymic index (Figure 5a). Histological analysis revealed that the thymus of μ-17-treated mice exhibited moderate reduction in cellularity throughout the experimental period and maintained cortical-medullary architecture (Figure 5b). μ-21 administration demonstrated remarkable prevention of thymic atrophy, with a 2.5-fold increase in thymic index observed on day 14 post-tumor inoculation, approaching values observed in healthy animals (Figure 5a). By day 18, the mean thymic index remained elevated, though increased inter-individual variability limited statistical significance. Histological analysis confirmed that μ-21 treatment effectively prevented thymic atrophy on day 14 but lost its positive effect on thymic structure by day 18 (Figure 5b). Combi treatment demonstrated significant inhibition of thymic involution, resulting in a statistically significant two-fold increase in thymic index by day 18 (Figure 5a). Notably, histological examination revealed that thymic architecture in mice receiving Combi was largely preserved and closely resembled that of healthy animals at both experimental time points (Figure 5b).

To assess the impact of miRNA-targeted oligonucleotide therapy on tumor-associated MDSC accumulation, we quantified M-MDSC (Supplementary Figure S6) and PMN-MDSC (Figure 5c,d) frequencies in the spleen and peripheral blood of CT-26 tumor-bearing mice following treatment. It was shown that M-MDSC levels were below 4% in both the peripheral blood and spleens of experimental animals (Supplementary Figure S6), while the accumulation of PMN-MDSCs was more substantial, reaching up to 60% in the blood and 10% in the spleen of tumor-bearing animals (Figure 5c,d). Analysis revealed that administration of µ-155 did not reduce splenic PMN-MDSC levels compared to the control group (Figure 5c). In contrast, µ-21 oligonucleotide showed a tendency to decrease splenic PMN-MDSCs by approximately 1.5-fold at an early time point (day 14), but this effect diminished by day 18 (Figure 5c). Conversely, µ-17 treatment exhibited a delayed but more pronounced effect, resulting in a significant four-fold reduction in PMN-MDSCs (*p* < 0.0001) by day 18 relative to the control (Figure 5c). Similarly, Combi therapy led to a modest reduction in PMN-MDSC levels at both day 14 and day 18, although these changes were not statistically significant (Figure 5c). Evaluation of circulating PMN-MDSCs in the peripheral blood on day 18 confirmed the observed immunomodulatory effects of µ-ONs. Consistent with the splenic data, µ-155 and µ-21 had no significant effect on PMN-MDSC frequencies in the blood (Figure 5d). In comparison, both µ-17 and Combi therapy effectively reduced blood PMN-MDSC levels by approximately two-fold and 2.2-fold, respectively, compared to controls (Figure 5d).

**Figure 6:**
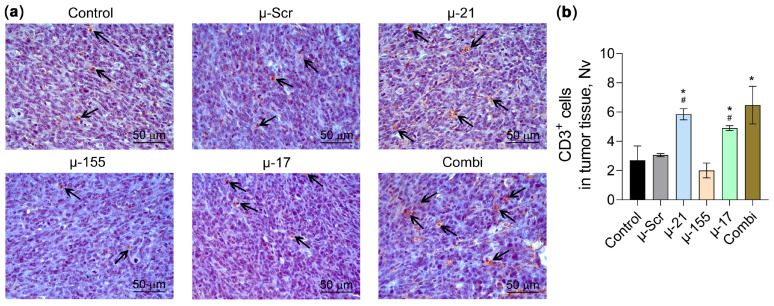
CD3^+^ T lymphocyte infiltration in CT-26 colorectal adenomas following miR-21, miR-17, and miR-155-targeted therapy. (**a**) Representative immunohistochemical images of adenomas stained with primary antibodies to CD3^+^ T-lymphocytes. Magnification ×400. Black arrows indicate CD3^+^ cells in tumor tissue. (**b**) Numerical density (Nv) of CD3^+^ T lymphocytes indicating the number of CD3^+^ cells in the square unit, 3.2 × 10^6^ μm^2^ in this case (*n* = 4). Data represent mean ± SEM. The data were analyzed using the Mann–Whitney U test. ^#^ and *—significant differences from control and μ-Scr, respectively, with *p* < 0.05.

To evaluate the impact of miRNA-targeted therapy on cytotoxic immune responses, we performed immunohistochemical analysis of CD3^+^ T lymphocyte infiltration in CT-26 adenomas on day 18 of tumor growth (Figure 6). Untreated control and µ-Scr-treated tumors demonstrated minimal CD3^+^ cell presence, amounting to 2.7 ± 1.0 and 3.1 ± 0.1 cells per test field, respectively (Figure 6a,b). Administration of μ-155 did not affect CD3^+^ T lymphocyte infiltration, whereas µ-21 and µ-17 treatment led to an increase in CD3^+^ cell density by 2.2- and 1.8-fold relative to control and 1.9- and 1.6-fold relative to µ-Scr. Combi treatment elicited the strongest T-cell immune response, increasing CD3^+^ lymphocyte infiltration by 2.4-fold compared to control and 2.1-fold compared to µ-Scr (Figure 6a,b).

Furthermore, serum concentrations of IFN-γ, IL-6, and TNF-α were measured to evaluate the systemic response to miRNA-targeted therapy. Unfortunately, no significant differences were found between treatment groups (Supplementary Figure S7).

The obtained results indicate the differential involvement of the investigated miRNAs in the immunoregulatory processes associated with thymic involution, tumor-associated MDSC accumulation and cytotoxic immune responses. The capacity to prevent thymic atrophy was observed with μ-21 and Combi therapy, whereas a reduction in tumor-associated PMN-MDSC expansion was achieved only with μ-17 and Combi therapy. Notably, CD3^+^ T lymphocyte infiltration of tumor tissue was comparably enhanced by μ-21, μ-17, and Combi therapy. These findings suggest that specific anti-miRNA therapy can simultaneously inhibit tumor growth, preserve immune organ function, restrict MDSC expansion, and restore cytotoxic anti-tumor immune response, potentially enhancing overall therapeutic efficacy through complementary mechanisms of action.

### 3.5. Effect of miRNA-Targeted Oligonucleotide Therapy on Survival in CT-26-Bearing Mice

**Figure 7:**
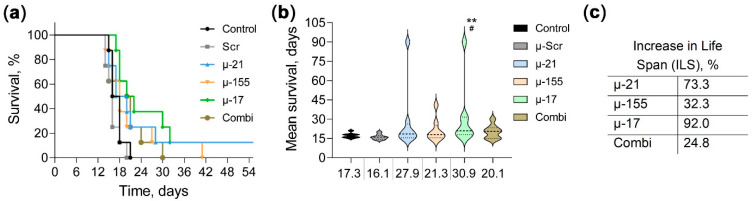
Effect of miR-21, miR-17, and miR-155-targeted therapy on the survival of mice with CT-26 colorectal carcinoma. (**a**) Kaplan–Meier survival curves of control and experimental groups (*n* = 8). (**b**) Mean survival time of mice in control and experimental groups. Data are presented as violin plots. Mean survival values for each group are indicated below the graph. ^#^—significant differences from control with *p* < 0.05; **—significant difference from μ-Scr with *p* < 0.01. (**c**) Increase in life span (ILS) of mice treated with miRNA-targeted oligonucleotides compared to the µ-Scr-treated group. ILS (%) = [(mean survival time of treated group − mean survival time of control group)/mean survival time of control group] × 100.

Concurrent with the assessment of anti-tumor and immunomodulatory effects of anti-miRNA therapy, survival analysis was performed to evaluate therapeutic efficacy. Mortality analysis in control and µ-Scr groups revealed near-complete lethality occurring between days 14 and 18 post-tumor transplantation. In contrast, mice receiving miRNA-targeted therapy demonstrated extended survival, with over half of the animals surviving beyond days 18–20. Notably, exceptional long-term survival (>90 days) was achieved by individual animals in both μ-21 and μ-17 groups. Kaplan–Meier survival curve analysis using the Log-rank (Mantel–Cox) test demonstrated statistically significant differences for μ-21, μ-17, and Combi groups compared to µ-Scr control, with μ-17 additionally showing significance versus untreated control (μ-21 vs. μ-Scr *p* = 0.047; μ-17 vs. μ-Scr *p* = 0.003 and vs. control *p* = 0.014; Combi vs. μ-Scr *p* = 0.034) (Figure 7a). Analysis of survival dynamics revealed that control and μ-Scr groups exhibited mean survival of 17.3 and 16.1 days, respectively (Figure 7b). μ-155 monotherapy and Combi treatment provided survival benefits, with mean survival times of 21.3 days (ILS 32.3%) and 20.1 days (ILS 24.8%), respectively (Figure 7b,c). μ-21 monotherapy demonstrated more pronounced therapeutic efficacy, achieving a mean survival time of 27.9 days, corresponding to a 73.3% increase in life span (Figure 7b,c). Remarkably, μ-17 treatment achieved statistically significant survival extension, with mean survival of 30.9 days, representing a 92.0% increase in life span and nearly doubling the lifespan compared to control groups (Figure 7b,c). These findings identify μ-17 as the most effective single-agent therapy in prolonging survival in the CT-26 peritoneal adenomatosis model.

## 4. Discussion

This study was designed to assess the potential of miRNA-targeted therapy to simultaneously suppress multiple tumor-associated processes in colorectal cancer. To address this issue, we used the CT-26 intraperitoneal adenomatosis model, characterized by highly aggressive growth kinetics and pronounced immunosuppression that creates a narrow therapeutic window for effective treatment. We demonstrate that miRNA-targeted therapy based on synthetic anti-sense oligonucleotides exerts target-dependent immunomodulatory anti-tumor effects.

Previous studies have demonstrated the therapeutic potential of miRNA inhibition in colorectal cancer [51]. LNA-anti-miR-21 inhibited cell growth, induced apoptosis and reduced invasive activity of LS174T cells in vitro [52]. Furthermore, a miR-21-targeting oligonucleotide, in addition to suppressing miR-21, downregulated miR-30 expression, resulting in reduced angiogenesis in HCT-116 cells [53]. Suppression of miR-675 decreased cell growth rate and colony formation in human colon cancer cells [54], while anti-miR-31 treatment increased sensitivity of HCT-116 cells to 5-FU [55]. In vivo, an miR-21 inhibitor delivered via engineered exosomes showed minimal effect as monotherapy, but significantly enhanced 5-fluorouracil efficacy in 5-FU-resistant HCT-116 colon cancer xenografts [56]. Moreover, iRGD-modified mesenchymal stem cell-derived exosomes loaded with anti-miR-221 oligonucleotides showed effective tumor site accumulation and notable anti-tumor activity [57]. Most significantly, the field has progressed to clinical evaluation, when good safety and a durable partial response lasting over three years were observed in one colorectal cancer patient during a phase I clinical trial of LNA-i-miR-221 (NCT04811898) [58]. Subsequent reverse translational studies confirmed that LNA-i-miR-221 reduces cell viability, induces apoptosis in several CRC cell lines, and inhibits HCT-116 tumor growth through the TP53 apoptotic pathway [59]. These findings highlight the translational promise of anti-miRNA approaches in colorectal cancer.

In our study, we investigated the therapeutic potential of highly nuclease-resistant mesyl oligonucleotides targeting miR-21, miR-155, and miR-17 in the CT-26 colorectal cancer model and revealed differential kinetics and efficacy of action among the investigated anti-miRs. Suppression of miR-155 showed minimal therapeutic impact throughout the study period, whereas miR-21 inhibition therapy achieved notable early-phase efficacy with two-fold tumor reduction, though this effect diminished at later timepoints. Most notably, miR-17-targeting treatment demonstrated a delayed but sustained response, achieving approximately 10-fold tumor growth inhibition at later stages. The combination therapy displayed stable activity with 3–4-fold tumor reduction maintained across early and late evaluation periods. These findings confirm the previously reported anti-tumor potential of miR-21 suppression in colorectal cancer and reveal the efficacy of miR-17-targeting therapy, showing distinct kinetics that suggest different underlying mechanisms of action.

The lower efficacy of the triple combination (Combi) compared to µ-17 monotherapy can be explained by suboptimal dosing of individual components. To maintain a total dose of 10 µg equivalent to monotherapy, each oligonucleotide was reduced three-fold to 3.3 µg per injection, which likely represents the minimal threshold for therapeutic activity. However, despite reduced magnitude of tumor suppression, Combi therapy showed earlier therapeutic response and more stable growth inhibition, suggesting complementary mechanisms of action of µ-21 and µ-17.

A remarkable and previously unreported finding is the preservation of thymic function by miR-21-targeting therapy during cancer progression. Interestingly, inhibition of miR-155 and miR-17 treatments had no effect on thymus function, even despite the strong anti-tumor effects of anti-miR-17. In contrast, miR-21-targeting and combination treatments provided significant thymus protection with more than a two-fold increase in thymic index. Thymic involution results from multiple causes including aging, infection, and cancer [49,50,60,61]. Accumulating evidence demonstrates that multiple miRNAs are involved in thymic development and pathology through post-transcriptional regulation of key genes [49,62,63,64]. While previous studies have largely concentrated on the role of intrathymic miRNA expression in thymic biology, the contribution of tumor-derived miRNAs to thymic function has received limited attention. Despite extensive research on miRNA-based anti-cancer therapeutics focusing on direct anti-tumor effects, no previous studies have demonstrated therapeutic preservation of thymic function through systemic anti-miRNA treatment in cancer. These results provide a new therapeutic concept: miRNA-targeted anti-cancer therapeutics can simultaneously inhibit tumor growth and preserve thymic function, potentially enhancing overall anti-tumor immunity through sustained T-cell production.

Furthermore, our findings confirm the critical role of miRNAs in tumor-associated immunosuppression and extend the current understanding of miRNA-targeted therapy in colorectal cancer. Previous studies have shown that miRNA inhibition can significantly modulate immunosuppression, though mechanisms may differ depending on the immune cellular context. In lung cancer, miR-21 inhibition via antagomir reduced MDSC level, increased Th and CTL populations, and decreased ARG-1 and iNOS expression [65]. However, it was demonstrated that targeted miR-21 inhibition in tumor-associated macrophages enhanced CD8^+^ T-cell cytotoxicity through increased IL-12 and CXCL10 production, though notably, they observed no changes in MDSC level [66].

In the CT-26 colorectal cancer model, we observed that therapeutic efficacy varied significantly depending on the specific miRNA target. Inhibition of miR-155 showed no significant effect on MDSC level. On the one hand, this is intriguing because miR-155 is known to be directly involved in enhancing the immunosuppressive properties and accumulation of MDSCs [25,29]. However, it is known that miR-155 acts pleiotropically, as a reduction in its levels can also promote MDSC accumulation [67,68]. Downregulation of miR-21 demonstrated a modest trend towards efficacy at early treatment stages. In contrast, miR-17-targeting and Combi therapy achieved approximately 50% MDSC reduction in both spleen and peripheral blood. This pronounced effect of miR-17 inhibition is particularly noteworthy, as the miR-17-92 cluster has been less extensively studied in the context of MDSC regulation compared to miR-21 and miR-155 [24,25], suggesting that miR-17 may represent an underappreciated target in colorectal cancer immunotherapy. The observed efficacy is quantitatively comparable to established MDSC-targeting agents such as ATRA [69] and sunitinib [70].

Survival studies provided additional evidence for the therapeutic value of miRNA-targeted therapy. Suppression of miR-17 provided the most significant survival extension, nearly doubling lifespan compared to control groups (mean survival 30.9 vs. 17.3 days, representing 92.0% ILS). miR-21-targeting monotherapy also demonstrated substantial efficacy with 73.3% ILS (27.9 days mean survival), while combination treatment achieved 24.8% ILS (20.1 days). The superior survival benefit of miR-17-targeting therapy correlates with its substantial anti-tumor activity and significant MDSC reduction at later treatment stages. The survival benefit of miR-21 silencing may be attributed to both its anti-tumor activity and unique thymic preservation that sustains T-cell function throughout disease progression. The more modest survival extension with combination therapy, despite stable anti-tumor activity, likely reflects reduced individual oligonucleotide doses, indicating that dose optimization may further improve therapeutic outcomes.

We propose that the observed therapeutic effects are primarily driven by miRNA inhibition within the tumor site, as supported by preferential oligonucleotide accumulation in adenoma tissue and target miRNA downregulation specifically in the tumor compartment. This tumor-directed miRNA suppression drives phenotypic changes that secondarily modulate the immune microenvironment. While our data support predominantly tumor-directed effects, contributions from other tissue compartments cannot be completely excluded.

An intriguing observation is the differential degree of effects seen at cellular versus organismal levels. Notably, µ-17 and Combi demonstrated superior in vivo therapeutic efficacy, revealing a dissociation between cellular-level and systemic effects where immunomodulatory mechanisms beyond direct cytotoxicity significantly contributed to therapeutic outcomes. The pronounced in vivo efficacy of µ-17 may be attributed to the associated reduction of MDSC populations, an effect not observed with µ-21 treatment, as well as to disruption of exosomal miR-17-mediated tumor-immune cross-talk. Exosomal miR-17 has been shown to play a prominent role in colorectal cancer immune evasion through suppression of T-cell infiltration and promotion of immunosuppressive cytokine production [71,72]. These results indicate that optimal anti-miRNA combinations should be selected based on complementary immunomodulatory mechanisms rather than solely on in vitro knockdown efficiency.

It is well known that the introduction of synthetic nucleic acids can induce nonspecific immune responses independent of their target-sequence effects. To assess the potential contribution of such effects to the observed therapeutic activity, we referred to our recent large-scale proteomic profiling of human colorectal adenocarcinoma Caco-2 cells after transfection with µ-ONs targeting miR-21, miR-155, and miR-17 [73]. This analysis demonstrated that 72 h post-transfection, miRNA-targeted µ-ONs (µ-17, µ-21, and µ-155), applied either individually or in triple combination, did not affect the levels of toll-like receptors (TLRs), their downstream adaptor MyD88, or interleukins, suggesting the absence of a non-specific innate immune response to the introduced synthetic oligonucleotides. However, a potential mechanism triggered by µ-ONs transfection appears to involve a cytosolic response to the oligonucleotides, as reflected in the modulation of DEAD-box helicases (DDX). In particular, analysis of the complete datasets of differentially expressed proteins in groups transfected with mono oligonucleotides (µ-17, µ-21, and µ-155) showed increased levels of DDX1, a protein directly implicated in the sensing of exogenous nucleic acids [74] (Table S2). Additionally, levels of DDX46, DDX18, and DDX42 were reduced across the µ-17, µ-21, µ-155, and Combi groups, suggesting suppression of transcriptional and translational processes within the tumor cells (Table S2) [75,76]. Importantly, this cytosolic response does not appear deleterious; on the contrary, it may potentiate the miRNA-mediated anti-tumor effects.

Taken together, our results demonstrate differential miRNA involvement in colorectal cancer progression and establish distinct therapeutic profiles for individual anti-miRNA agents. miR-21 inhibition provides early-stage anti-tumor effect and simultaneously preserves immune organ function, extending survival. miR-17 inhibition therapy demonstrates delayed but substantial anti-tumor activity, significantly inhibits immunosuppressive mechanisms, and achieves the most pronounced survival extension. The combination approach integrates the individual therapeutic advantages of both agents, resulting in more stable anti-tumor efficacy, thymic protection, immunosuppression control, and enhanced survival outcomes. While our previous studies demonstrated efficacy of the triple combination in melanoma and lymphosarcoma models [11], the CT-26 colorectal cancer model revealed tumor type-specific response, with minimal therapeutic contribution from µ-155. Given the distinct therapeutic profiles observed and the minimal contribution of µ-155, a dual combination of µ-17 and µ-21 would likely represent a more optimal strategy. Future studies employing dose optimization, refined administration schedules, and evaluation of the µ-17/µ-21 dual combination may reveal superior therapeutic outcomes compared to either monotherapy or the current triple combination regimen.

Overall, these findings establish anti-miRNA therapy as a promising multitargeted approach for colorectal cancer treatment that simultaneously addresses tumor burden, immune organ integrity, and systemic immunosuppression.

### Limitations of the Study

This study has several limitations. First, we utilized a single tumor model (CT-26 peritoneal carcinomatosis) in female mice only, which may limit generalizability across tumor types, sexes, and immune contexts. Second, while enhanced CD3^+^ T lymphocyte infiltration was observed following treatment, comprehensive characterization of T-cell subsets (CD8^+^ and CD4^+^ T-cells, Tregs) and their functional properties (expression of perforin, granzime B, IFNγ, PD-1) require further investigation. Third, oligonucleotide dosing and administration schedules were suboptimal due to the biphasic tumor growth kinetics observed in this model, optimization of dosing strategies aligned with tumor growth phases may further enhance therapeutic efficacy and improved survival outcomes. Fourth, although we observed effects on tumor-associated MDSC populations and thymic preservation, direct mechanistic studies examining oligonucleotide-mediated changes in MDSC phenotype/function and thymic cellular composition require further investigation.

## Data Availability

The data generated or analyzed during this study are included in this article or Supplementary Materials accompanying this paper. Further inquiries can be directed to the corresponding author.

## Supplementary Materials

The following supporting information can be downloaded at: https://www.mdpi.com/article/10.3390/pharmaceutics18010122/s1, Table S1: RT and PCR primers; Figure S1: Gating strategy for the identification of mouse MDSC subsets; Figure S2: Myeloid-derived suppressor cells (M-MDSCs) during CT-26 progression; Figure S3: Immune cell populations within CT26 adenomas; Figure S4: Representative photographs of the abdominal cavity in mice treated with individual or combined miRNA-targeted oligonucleotides; Figure S5: Validation of miRNA suppression and target protein upregulation in adenomas; Figure S6: Immunomodulatory effects of miR-21, miR-17, and miR-155-targeted therapy on M-MDSC populations; Figure S7: Cytokine concentrations in blood serum of mice on day 18; Table S2: Differential expression of DEAD-box helicases (DDX) in Caco-2 cells after treatment with μ-ONs targeted to miR-21, miR-155, and miR-17 relative to the control intact Caco-2 cells. Results are from proteomic profiling conducted in the research described in [73].

## Abbreviations

The following abbreviations are used in this manuscript:

2X3-DOPE	1,26-bis(cholest-5-en-3β-yloxycarbonylamino)-7,11,16,20-tetraazahexacosane-dioleoylphosphatidylethanolamine
5-FU	5-fluorouracil
AKT	protein kinase B
Arg1	arginase-1
ATRA	all-trans retinoic acid
CRC	colorectal cancer
CTL	cytotoxic T lymphocytes
CXCL10	C-X-C motif chemokine ligand 10
E2F	E2F transcription factor
IL-10	interleukin-10
IL-12	interleukin-12
ILS	increase in lifespan
iNOS	inducible nitric oxide synthase
iRGD	internalizing RGD
LNA	locked nucleic acid
MAPK	mitogen-activated protein kinase
MDSC	myeloid-derived suppressor cell
MLH1	MutL homolog 1
MSH2	MutS homolog 2
MSH6	MutS homolog 6
MTT	3-(4,5-dimethylthiazol-2-yl)-2,5-diphenyltetrazolium bromide
PDCD4	programmed cell death 4
PI3K	phosphoinositide 3-kinase
PMN-MDSC	polymorphonuclear myeloid-derived suppressor cells
PTEN	phosphatase and tensin homolog
RBC	red blood cell
RND3	Rho family GTPase 3
RORA	retinoic acid receptor-related orphan receptor alpha
SMAD7	mothers against decapentaplegic homolog 7
SOCS-1	suppressor of cytokine signaling 1
TGF-β	transforming growth factor beta
Th	T helper cells
TP53	tumor protein p53
