# Supplementary material for: Targeted Inhibition of Oncogenic microRNAs miR-21, miR-17, and miR-155 Suppresses Tumor Growth and Modulates Immune Response in Colorectal Cancer
Source: Pharmaceutics. 2026 Jan 18;18(1):122. doi: 10.3390/pharmaceutics18010122 (PMC12845401; doi:10.3390/pharmaceutics18010122)
Supplement: Supplementary file 1 [file pharmaceutics-18-00122-s001.zip › pharmaceutics-4003386-supplementary.pdf]

SUPPLEMENTARY MATERIALS

Article

Targeted Inhibition of Oncogenic microRNAs miR-21, miR-17 and miR-155 Suppresses Tumor Growth and Modulates Immune Response in Colorectal Cancer

Olga Patutina[1], Aleksandra Sen'kova[1], Svetlana Miroshnichenko[1], Mona Awad[1,2], Oleg Markov[1], Daniil Gladkikh[1], Innokenty Savin[1], Ekaterina Seroklinova[1], Sergey Zhukov[1], Maxim Kupryushkin[1], Mikhail Maslov[3], Valentin Vlassov[1] and Marina Zenkova[1,*]

1 Institute of Chemical Biology and Fundamental Medicine, SB RAS, Novosibirsk, Russia;
2 Novosibirsk State University, Novosibirsk, Russia;
3 MIREA – Russian Technological University, Moscow, Russia.

Table S1. RT and PCR primers used in the study.

Name	Type	Sequence (5' → 3')
RT-miR-21	RT	GTCGTATCCAGTGCAGGGTCCGAGGTATTCGCACTGG ATACGACTCAACATCAG
RT-miR-155	RT	GTCGTATCCAGTGCAGGGTCCGAGGTATTCGCAC TGGATACGACGACACCCCTATCA
RT-miR-17	RT	GTCGTATCCAGTGCAGGGTCCGAGGTATTCGCAC TGGATACGACCTACCTGCAC
RT-U6	RT	GTCGTATCCAGTGCAGGGTCCGAGGTATTCGCACTGG ATACGACAAAAATATGGAACCC
RT-SNORD43	RT	GTCGTATCCAGTGCAGGGTCCGAGGTATTCGCACTGG ATACGACAATCAGAACTTGAC
miR-21-F	PCR	AGACTAGCTTATCAGACTGA
mir-155-F	PCR	ACTTAATGCTAATTGTGATAGG
mir-17-F	PCR	AGACAAAGTGCTTACAGTGC
U6-F	PCR	CTCGCTTCGGCAGCACA
SNORD43-F	PCR	GACGGGCGGACAGAAACT
Universal-R	PCR	GTGCAGGGTCCGAGGT

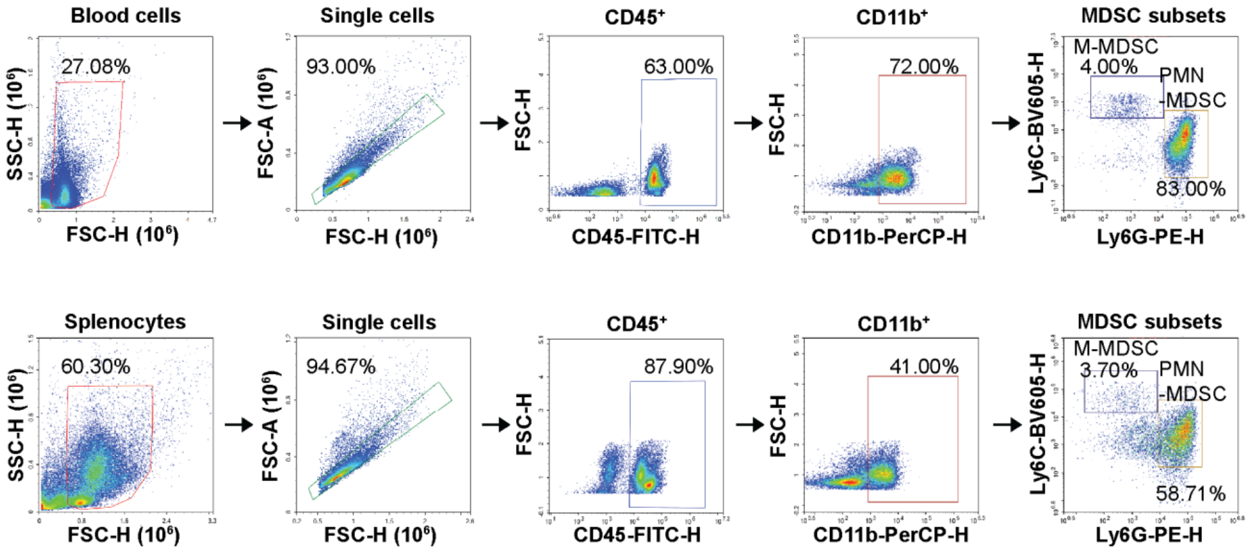

Figure S1. Gating strategy for the identification of mouse MDSC subsets in the peripheral blood and spleen of CT-26 tumor-bearing mice.

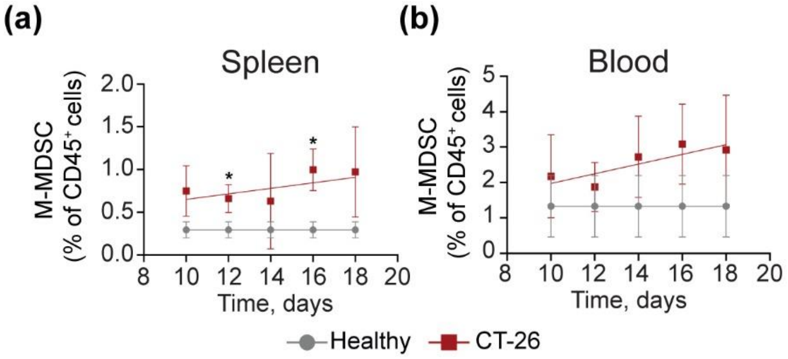

Figure S2. The levels of monocytic MDSCs (M-MDSCs) during CT-26 colorectal carcinoma progression. Percentage of $CD11b^{+}Ly6G^{-}Ly6C^{high}$ M-MDSCs in (**a**) spleen and (**b**) peripheral blood. Data were analyzed using one-way ANOVA followed by Tukey's post hoc test. Values represent mean ± SEM (n = 4–5). * – $p < 0.05$ vs healthy control.

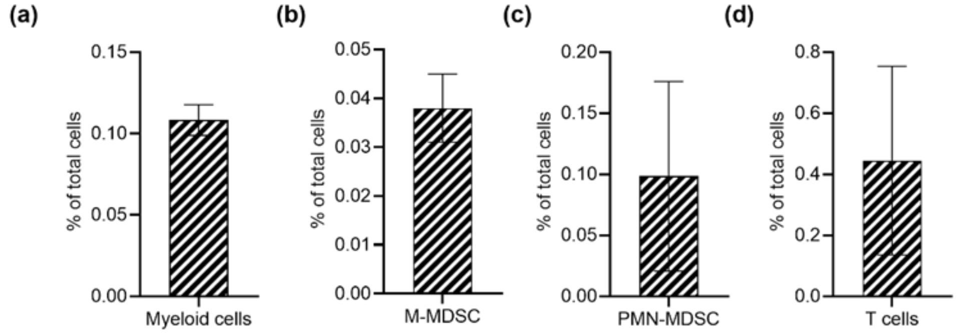

Figure S3. CT-26 tumor infiltration by immune cell populations on day 18 after tumor transplantation. (**a–d**) Percentage of immune cells among total tumor cells (**a**) $CD45^{+}CD11b^{+}$ myeloid cells, (**b**) $CD45^{+}CD11b^{+}Ly6G^{-}Ly6C^{high}$ M-MDSCs, (**c**) $CD45^{+}CD11b^{+}Ly6G^{+}Ly6C^{low}$ PMN-MDSCs, and (**d**) $CD45^{+}CD3^{+}$ T cell. Data are presented as mean ± SD ($n = 4$-5).

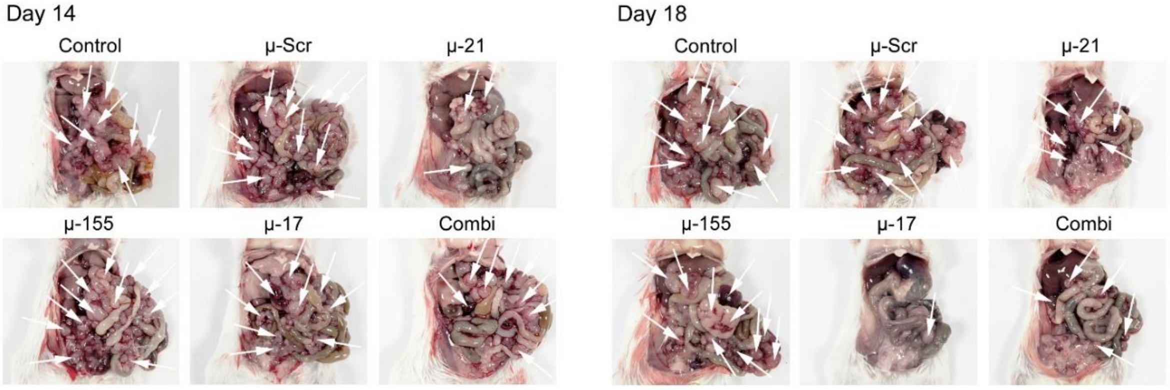

Figure S4. Representative photographs of the abdominal cavity in mice treated with individual or combined miRNA-targeted oligonucleotides on days 14 and 18 of CT-26 peritoneal adenomatosis progression. Arrows indicate adenoma lesions.

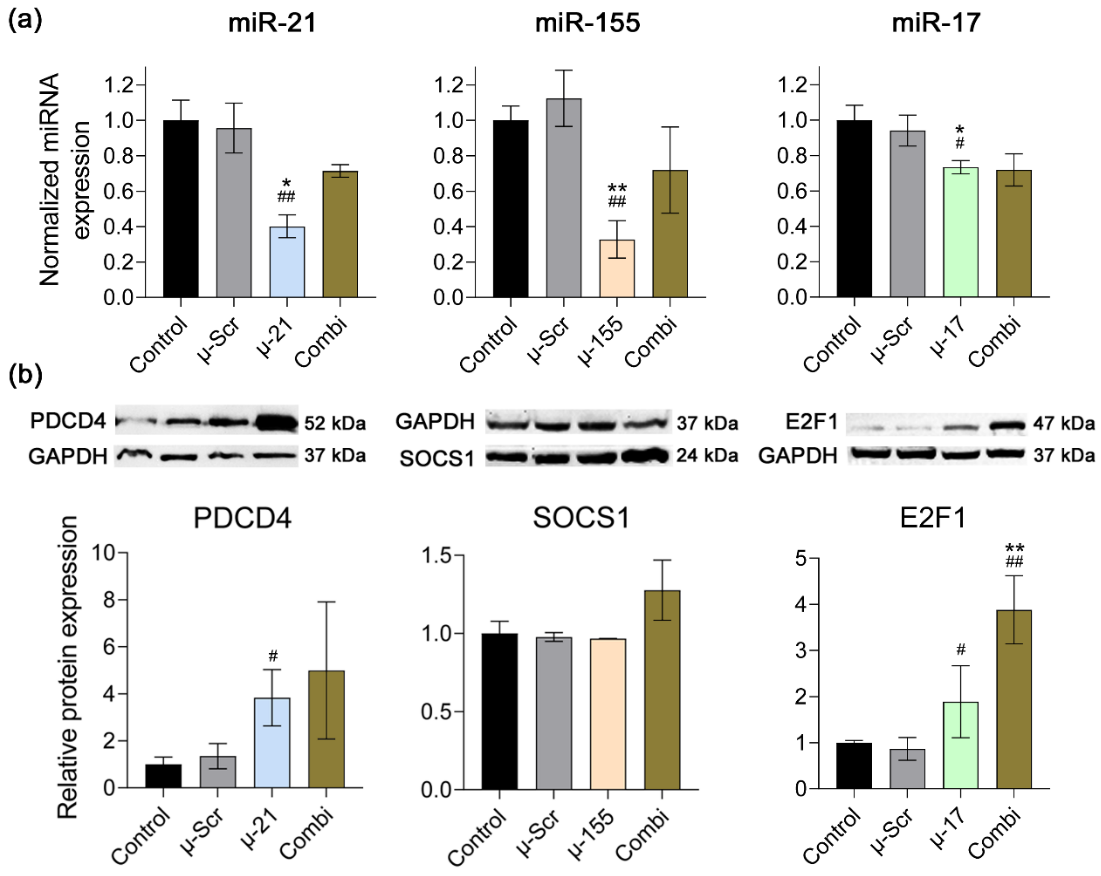

Figure S5. Validation of target miRNA suppression and downstream protein upregulation in adenoma tissue. (**a**) Relative expression of miR-21, miR-155, and miR-17 in adenoma tissues after μ-ONs treatment. miRNA levels were determined by stem-loop RT-qPCR and normalized to U6 and SNORD43 as dual reference controls. (**b**) Relative protein levels of PDCD4, SOCS1 and E2F1 proteins, representing the direct targets of miR-21, miR-155, and miR-17, respectively, in adenoma tissues after μ-ONs treatment. Protein level was normalized on the level of housekeeping protein GAPDH. PCR and Western blot analysis were carried out at the third day after the fourth injection (day 18 of tumor growth). Data were analyzed using Student's t-test. $^{\#}$, $^{\#\#}$—significant differences from Control with *p*<0.05, and *p*<0.01. *, and **—significant differences from μ-Scr with *p*<0.05, and *p*<0.01, respectively.

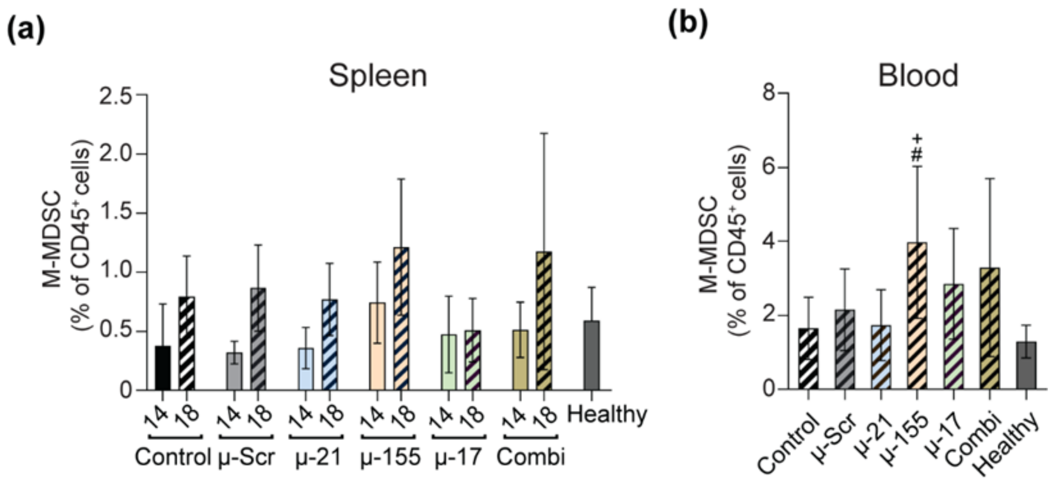

Figure S6. Immunomodulatory effects of miR-21, miR-17, and miR-155-targeted therapy on **M-MDSC** populations in the CT-26 colorectal cancer model. (**a**) Percentage of M-MDSCs in spleen on days 14 (solid bars) and 18 (striped bars). (**b**) Percentage of M-MDSCs in peripheral blood on day 18. Data represent mean ± SEM (*n*=4). The data were analyzed using one-way ANOVA followed by Tukey's post hoc test. $^{\#}$–$p < 0.05$ vs control. $^{+}$–$p < 0.05$ vs healthy.

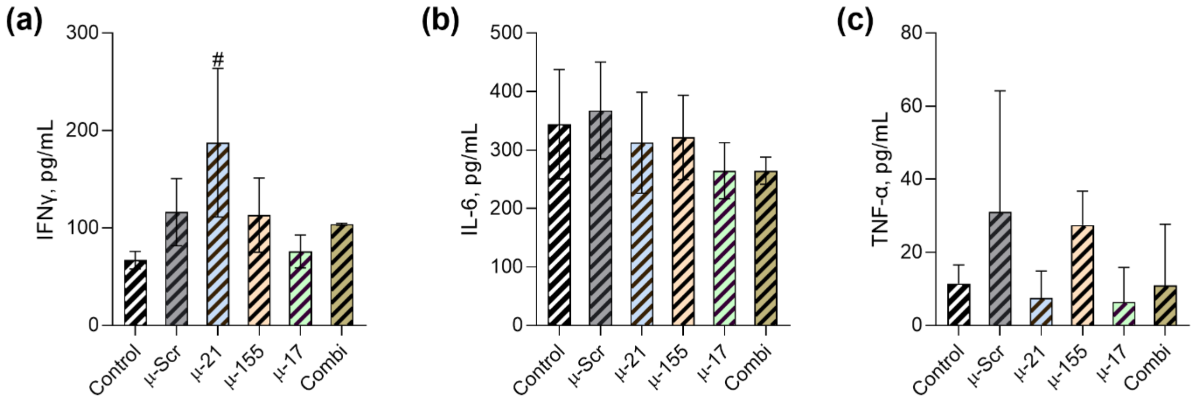

Figure S7. Effects of miR-21, miR-17, and miR-155-targeted therapy on the blood cytokines on day 18 after CT-26 tumor transplantation. The blood levels of (**a**) IFN-γ, (**b**) IL-6, and (**c**) TNF-α. Data represent mean ± SD (n = 4). Data were analyzed using one-way ANOVA followed by Tukey's post hoc test. $^{\#}$–$p < 0.05$ vs control.

Table S2. Differential expression of DEAD-box helicases (DDX) in Caco-2 cells after treatment with μ-ONs targeted to miR-21, miR-155, and miR-17 relative to the control intact Caco-2 cells. Results are from proteomic profiling conducted in the research described in [73].

**DDX**	**μ-ON-treated group**	**Log_2(Fold Change)**	**Function of DDX in cells**
DDX1	μ-21	1.16	Response to the synthetic oligonucleotide
	μ-17	1.32	
	μ-155	1.48	
DDX18	μ-21	–2.22	Regulation of transcription and translation, intracellular transport
	μ-155	–1.41	
DDX42	μ-17	–1.59	
	Combi	–1.90	
DDX46	μ-17	–0.80	
	μ-155	–1.56	
	Combi	–1.90	

73. Miroshnichenko, S.K.; Patutina, O.A.; Markov, A.V.; Kupryushkin, M.S.; Vlassov, V.V.; Zenkova, M.A. Biological Performance and Molecular Mechanisms of Mesyl MicroRNA-Targeted Oligonucleotides in Colorectal Cancer Cells. Int. J. Mol. Sci. 2025, 26, 11747. https ://doi.org/10.3390/ijms262311747.
